# Broccoli (*Brassica oleracea* L. var. *italica*) Sprouts as the Potential Food Source for Bioactive Properties: A Comprehensive Study on In Vitro Disease Models

**DOI:** 10.3390/foods8110532

**Published:** 2019-10-30

**Authors:** Thanh Ninh Le, Hong Quang Luong, Hsin-Ping Li, Chiu-Hsia Chiu, Pao-Chuan Hsieh

**Affiliations:** Department of Food Science, National Pingtung University of Science and Technology, Pingtung 91207, Taiwan; ltninh90@gmail.com (T.N.L.); lhquang@hcmuaf.edu.vn (H.Q.L.); c8976754@gmail.com (H.-P.L.); cschiou@mail.npust.edu.tw (C.-H.C.)

**Keywords:** sprouts, antioxidant activity, antiproliferative activity, apoptosis, antibacterial activity

## Abstract

Broccoli sprouts are an excellent source of health-promoting phytochemicals such as vitamins, glucosinolates, and phenolics. The study aimed to investigate in vitro antioxidant, antiproliferative, apoptotic, and antibacterial activities of broccoli sprouts. Five-day-old sprouts extracted by 70% ethanol showed significant antioxidant activities, analyzed to be 68.8 μmol Trolox equivalent (TE)/g dry weight by 2,2′-azino-bis-3-ethylbenzothiazoline-6-sulphonic (ABTS) assay, 91% scavenging by 2,2-diphenyl-1-picrylhydrazyl (DPPH) assay, 1.81 absorbance by reducing power assay, and high phenolic contents by high-performance liquid chromatography (HPLC). Thereafter, sprout extract indicated considerable antiproliferative activities towards A549 (lung carcinoma cells), HepG2 (hepatocellular carcinoma cells), and Caco-2 (colorectal adenocarcinoma cells) using 3-(4,5-dimethylthiazol-2-yl)-2,5-diphenyltetrazolium bromide (MTT) assay, with IC_50_ values of 0.117, 0.168 and 0.189 mg/mL for 48 h, respectively. Furthermore, flow cytometry confirmed that Caco-2 cells underwent apoptosis by an increase of cell percentage in subG1 phase to 31.3%, and a loss of mitochondrial membrane potential to 19.3% after 48 h of treatment. Afterward, the extract exhibited notable antibacterial capacities against *Bacillus subtilis* and *Salmonella* Typhimurium with minimum inhibition concentration (MIC) values of 0.39 and 0.78 mg/mL, appropriately, along with abilities against *Staphylococcus aureus* and *Escherichia coli* with an MIC value of 1.56 mg/mL. Thus, broccoli sprouts were confirmed as a potential food source for consumers’ selection and functional food industry.

## 1. Introduction

In recent years, increasing attention has been given to fruits and vegetables in the context of healthy diets because they are good sources of nutrients, dietary fiber, minerals, carotenoids, and phenolic compounds [1,2]. Numerous studies have demonstrated that the regular consumption of plant-based food may reduce the risk of developing chronic health conditions, including cardiovascular diseases and different types of cancer [3,4]. However, fresh fruits and vegetables are generally seasonal and thus most of the out-of-season crops are cultivated under unnatural conditions. These growing conditions (soil, compost, hydroponic) directly affect plant growth, as well as nutrient and mineral composition. An excellent alternative for plant foods are sprouts, which can be consumed in fresh form throughout the year. Moreover, sprout cultivation could reduce the use of fertilizer and pesticides [5,6]. Edible sprouts are thought to be rich in health-promoting phytochemicals that are known to prevent a number of chronic and degenerative diseases. Common sprouting seeds include mung beans, radish, broccoli, sunflower, soybean, cabbage, wheat, rice, and others [5,6]. Recently, a great deal of attention in the food industry has been given to natural plant products due to their immune-strengthening potential and the ability to prevent many of today’s most common diseases [7,8]. Thus, edible sprouts may be utilized in functional food products with the aim of enhancing the quality and nutritive value of foods.

Broccoli (*Brassica oleracea* L. var. *italica*) sprouts have gained recognition as nutraceutical foods due to the rich presence of bioactive compounds, including glucosinolates, polyphenols, carotenoids, minerals, and vitamins, with concentrations higher than the adult plant [9,10,11]. These defense-related secondary metabolites have been positively linked to different biological capacities, such as anti-diabetic, anticarcinogenic, anti-inflammatory, and antioxidant properties [10,12]. Particularly, anticancer and antioxidant activities of broccoli sprouts have attracted the most attention, and have been extensively studied over the last years. To demonstrate the cancer prevention effect of broccoli sprouts, most of the investigations examined its glucosinolate profile, and cytotoxic activity against different in vitro carcinogenesis models [10,13,14]. Nonetheless, to date, the detailed studies which investigated the mechanism of cancer cell death induced by broccoli sprout extract such as apoptotic, autophagic, or cell cycle arrest effects, have not been well documented [7,13]. Additionally, to the best of our knowledge, only a few publications have focused on antimicrobial activity of broccoli florets and stems [15,16,17], however no reports have yet been specified on antibacterial properties of broccoli sprouts against food spoilage and food pathogenic bacteria.

Towards this end, the objective of the present work was to extensively characterize bioactivities of broccoli sprouts on various in vitro models, including the antioxidant, anticancer and antibacterial potential. Notably, this study aimed to highlight that the antiproliferative effect of broccoli sprouts might be associated with inducing apoptosis, and that it is the first to demonstrate the detrimental effect of broccoli sprouts on foodborne pathogens.

## 2. Materials and Methods

### 2.1. Plant Material and Cultivation Condition

Broccoli seeds (*Brassica oleracea* L. var. *italica*) were purchased from Known-You Seed Ltd. (Kaohsiung, Taiwan). They had been stored under vacuum at low temperature and a preliminary test revealed that their germination performance was very good, with a yield rate of approximately 95%.

Broccoli seeds were sterilized by immersion in 70% ethanol (5 g/L) for 1 min, followed by immersion in 1.5% sodium hypochlorite (NaOCl) for 15 min (min). Ethanol and NaOCl were removed with three rinses of deionized water and drained off. Seeds were then steeped in deionized water in ratio 1:10 (*m*/*v*) for 12 h (h). After pouring off the soaking water, each 5 g of seeds, corresponding to over 1000 seeds, were weighed and spread evenly on trays (26 × 20 × 4 cm) lined with moistened paper towels and filter papers, and irrigated six times a day using deionized water to guarantee constant water availability. After seeds were initially peeled off their shells, the trays were transferred to a controlled environment chamber at air temperature of 25 °C and a photoperiod regime with cycles of 16 h light and 8 h darkness. Sprouts were collected after 3, 5, 8, 10, and 12 days of germination [5,18].

After harvesting, sprouts were washed with running tap water and rinsed with deionized water, then dried at 40 °C for 24 h using an electric drying oven (Nabertherm GmbH, Lilienthal, Germany). All samples were ground to a fine powder, and stored at −20 °C until further analysis.

### 2.2. Crude Extract Preparation

For antioxidant analysis, samples were extracted in different selected solvents. Briefly, broccoli sprout powder (10 g) was added to 70% methanol, 70% ethanol, and hot water (100 mL) to reach the concentration of 0.1 g dry weight (DW)/mL. Ethanol and methanol extractions were performed by an orbital shaker for 24 h at 20 °C. Hot water extraction was carried out by adding the sample into boiling deionized water and continuous stirring for 15 min. All extracts were centrifuged at 10,000 rpm for 10 min and the supernatants were filtered by Whatman No. 1 filter paper [18,19]. Filtrates were then directly used for antioxidant determination.

For anticarcinogenic and antibacterial experiments, the filtrates were evaporated to dryness using a rotary vacuum evaporator (RV8, IKA Works Guangzhou, Guangzhou, China) at 35–40 °C, and then freeze-dried (Martin Christ GmbH, Osterode, Germany). The lyophilized broccoli sprout extract (BSE) was stored at −20 °C until further analysis.

### 2.3. Determination of Total Phenolic Content (TPC)

Samples (100 μL, 0.1 g DW/mL) were added to deionized water (1 mL) and Folin–Ciocalteau reagent (200 μL, 2N). After 3 min at 25 °C, sodium carbonate solution (1.5 mL, 20%) was added. The reaction mixture was incubated for 90 min at the same temperature. Absorbance was then measured at 765 nm using a microplate plate reader (Thermo Fisher Scientific, Waltham, MA, USA). Gallic acid standards in the range of 0–100 ppm were treated in a similar manner to generate a calibration curve (r^2^ = 0.9959) to calculate TPC [8,20]. The TPC of the extracts was expressed as gallic acid equivalent (GAE) g/100 g DW.

### 2.4. Determination of Total Flavonoid Content (TFC)

Samples (0.25 mL, 0.1 g DW/mL) were mixed with sodium nitrite solution (75 μL, 5%), aluminum trichloride solution (0.15 mL, 10%), and sodium hydroxide solution (0.5 mL, 1 M). The final volume of the mixture was adjusted to 2.5 mL with deionized water, and allowed to incubate for 5 min at 30 °C. The absorbance was measured at 490 nm. Catechin standards in the range of 0–100 ppm were treated in a similar manner to generate a calibration curve (r^2^ = 0.9975) to calculate TFC [8,20]. The TFC of the extracts were expressed as catechin equivalent (CE) g/100 g DW.

### 2.5. Determination of Vitamin C Content

Samples (0.5 mL, 0.1 g DW/mL) were added to trichloroacetic acid (0.8 mL, 10%) and vigorously shaken. The mixture was kept on ice for 5 min and centrifuged at 3000 rpm for 5 min. The mixture (0.2 mL) was diluted with deionized water (2 mL). The commercially prepared Folin–Ciocalteu (2.0 M) was diluted 10-fold before it (0.2 mL) was added to the mixture, and allowed to stand for 10 min at 30 °C. Absorbance was measured at 760 nm. Ascorbic acid standards in the range of 0–100 ppm were treated in a similar manner to generate a calibration curve (r^2^ = 0.9953) to calculate vitamin C content [21,22]. The vitamin C content was expressed as ascorbic acid equivalent (AA) g/100 g DW.

### 2.6. 2,2-Diphenyl-1-Picrylhydrazyl (DPPH) Radical Scavenging Assay

Samples (5 mL, 0.1 g DW/mL) and standard compound of ascorbic acid solutions (5 mL, 0.5 mg/mL) were separately mixed with freshly prepared DPPH (2,2-diphenyl-1-picrylhydrazyl) methanolic solution (1 mL, mM). The reaction mixture was vortexed and allowed to stand in the dark at 30 °C for 60 min. The absorbance of samples and standard was recorded against a blank at 517 nm. The percentage of the DPPH scavenging activity was calculated using the following formula: % inhibition of DPPH radical = [(absorbance of blank − absorbance of sample)/absorbance of blank] × 100% [8,23].

### 2.7. 2,2′-Azino-Bis-3-Ethylbenzothiazoline-6-Sulphonic (ABTS) Radical Cation Decolorization Assay

ABTS (2,2′-azino-bis-3-ethylbenzothiazoline-6-sulphonic) was dissolved in water to make a concentration of 7 mmol/L. ABTS⁺ was produced by mixing the ABTS stock solution with potassium persulfate (2.45 mmol/L) and allowing the mixture to stand in the dark at room temperature for 12–16 h before use. Then the ABTS⁺ stock solution was diluted with PBS (5 mmol/L, pH 7.4) to an absorbance of 0.70 (±0.02) at 734 nm and equilibrated at 30 °C for 30 min. Samples (10 μL, 0.1 g DW/mL) were added to diluted ABTS⁺ solution (1 mL), and absorbance reading at 734 nm was taken 5 min after the initial mixing. Trolox standards in the range of 0–100 ppm were treated in a similar manner to generate a calibration curve (r^2^ = 0.9958) [23,24]. The antioxidant capacity of the extracts was expressed as Trolox equivalent (TE) μmol/100 g DW.

### 2.8. Reducing Power Assay

Samples (5 mL, 0.1 g DW/mL) accompanied with ascorbic acid solution (5 mL, 0.5 mg/mL) were spiked separately with PBS (phosphate-buffered saline) solution (2.5 mL, 0.2 M, pH 6.6) and potassium ferricyanide solution (2.5 mL, 1%). The mixture was incubated at 50 °C for 20 min, then added with a trichloroacetic acid solution (2.5 mL, 10%), and centrifuged at 3000 rpm for 10 min. The supernatant (2.5 mL) was mixed with deionized water (2.5 mL) and iron (III) chloride solution (0.5 mL, 0.1%), and allowed to stand for 10 min. The absorbance was recorded at 700 nm against a blank [23,25]. Higher absorbance indicated higher reducing power.

### 2.9. High-Performance Liquid Chromatography (HPLC) Analysis

Ethanolic extracts (20 μL), previously filtered using a 0.45 μm nylon membrane filter, were injected in the HPLC system (Hitachi Chromaster, Tokyo, Japan). The chromatographic separation was carried out with a NUCLEODUR^®^ C_18_ HTec column (250 × 4.6 mm, the particle size of 5 μm, Macherey-Nagel, Düren, Germany). The mobile phase consisted of deionized water containing 0.1% trifluoroacetic acid (A) and methanol (B). The gradient was programmed in the following order: 90% A at 0–3 min, 70% A at 20 min, 60% A at 30 min, 40% A at 50 min, and 80% A at 60 min. The flow rate was 1.0 mL/min, and the column temperature was maintained at 25 °C. Phenolic compounds were detected by comparing their chromatographic behavior and monitoring UV absorption at 320 nm with authentic standards and reported data [5,10,19].

### 2.10. Cell Culture

The following cell lines used for the present study were obtained from the Food Industry Research and Development Institute, Hsinchu, Taiwan. HepG2 (hepatocellular carcinoma cells) and Caco-2 (colorectal adenocarcinoma cells) were cultured in the DMEM medium. A549 (lung carcinoma cells) and FL83B (normal liver cells) were cultured in F-12 medium. The growth media was supplemented with 10% fetal bovine serum (FBS), penicillin (100 U/mL) and streptomycin (100 μg/mL) and sodium bicarbonate (1.5 g/L). All cells were maintained at 37 °C in a humidified 5% CO_2_ atmosphere.

### 2.11. Proliferation Assay

Cells (1 × 10^4^ cells/200 μL/well) were seeded into 96-well microplates for 24 h of cell adherence. Cells were then treated separately with medium alone, broccoli sprout extract (0.063–0.500 mg/mL) and cisplatin (0.006–0.050 mg/mL) at different concentration ranges for 24 and 48 h. Cisplatin, a conventional chemotherapeutic drug, was used as a positive control. MTT (3-(4,5-dimethylthiazol-2-yl)-2,5-diphenyltetrazolium bromide) solution (10 μL, 5 mg/mL) was then added per well and incubated for 2 h. After the medium was removed, the formed formazan crystals were dissolved in DMSO (100 μL). Absorbance was measured at 570 nm (with reference at 620 nm) in an ELISA plate reader (Thermo Fisher Scientific, Waltham, MA, USA). The percentage cell viability was determined by using the following formula: % cell viability = (absorbance of sample/absorbance of control) × 100% [20,26]. The half-maximal inhibitory concentration (IC_50_) for extracts was determined using Graphpad Prism 5 software.

### 2.12. Cell Cycle Analysis

Caco-2 cells (1 × 10^6^ cells) were harvested 24 and 48 h after separately adding medium alone, extracts (0.200 mg/mL) and cisplatin (0.020 mg/mL), washed in PBS, fixed in cold 70% ethanol and kept at −20 °C overnight. Fixed cells were washed again in PBS and re-suspended in PI solution (1 mL) containing propidium iodide (0.050 mg/mL), Triton X-100 (0.5%) and RNAse A (0.050 mg/mL), then incubated in the dark for 30 min at 37 °C. Cells were analyzed for cell cycle distribution at different phases (G0/G1, S, G2/M, and subG1) in the FACS Calibur flow cytometer (BD Biosciences, San Jose, CA, USA) [7,24].

### 2.13. Measurement of Mitochondrial Membrane Potential (MMP)

Caco-2 cells (1 × 10^6^ cells) were harvested 3, 6, 12, 24, and 48 h after adding medium alone, extracts (0.200 mg/mL) and cisplatin (0.002 mg/mL), then washed in PBS, incubated with DiOC_6_ (3,3′-dihexyloxacarbocyanine iodide) solution (1 mL, 0.5 μg/mL) in the dark for 30 min at 37 °C, and re-suspended in PBS (1 mL). Retained DiOC_6_ concentration, which represented the changes in MMP in treated cells, was analyzed by FACS Calibur flow cytometer (BD Biosciences, San Jose, CA, USA) [27,28].

### 2.14. Bacterial Culture

Four bacterial strains were selected for the antibacterial tests, including the Gram-positive bacteria *Staphylococcus aureus* CCRC 11863 (Culture Collection and Research Center, Hsinchu, Taiwan) and *Bacillus subtilis* CCRC 14199, and Gram-negative bacteria *Salmonella* Typhimurium CCRC 12497 and *Escherichia coli* CCRC 11634. *S. aureus* and *E. coli* were cultured in Tryptic soy broth, and *B. subtilis* and *S.* Typhimurium were cultured in Nutrient broth at 37 °C for 18 h to obtain subcultures.

### 2.15. Agar Diffusion Method

The agar well diffusion method was employed for the determination of the antimicrobial activity of the extracts. Wells were made in nutrient agar plates using cork borer (9 mm diameter), previously inoculated with culture suspension (1%, 1 × 10^6^ CFU/mL). Each 100 μL of sample extract that was diluted in DMSO (20%) to a concentration of 50 mg/mL, and negative control (20% DMSO) or standard antibiotics including ampicillin and amoxicillin (0.1 mg/mL) was separately filled in wells. The plates were then incubated under suitable conditions for the tested bacteria at 37 °C for 16 h. The diameter (mm) for the zone of inhibition was measured using calipers [29,30].

### 2.16. Determination of the Minimum Inhibition Concentration (MIC)

The broth microdilution method was performed for MIC determination. The procedure involved preparing various two-fold dilutions of extract (0.1–50 mg/mL) in a liquid growth medium dispensed in 96-well microtitration plate. Ampicillin (0.1 mg/mL) was used as a positive control. Each well was inoculated with culture suspension after dilution to match 1 × 10^6^ CFU/mL. The inoculated 96-well plate was incubated at 37 °C for 16 h. MIC values (mg/mL) were detected as the minimum concentration where microbial growth was missing. MTT (20 μL, 5 mg/mL) was added to each well as an indicator of microbial growth and observed for color development after 2 h [29,31,32].

### 2.17. Statistical Analysis

For statistical analysis, SPSS 22.0 software was used. All experiments were performed in three replications (*n* = 3). Data were expressed as means ± standard deviation (SD) and studied using a one-way analysis of variance (ANOVA). When ANOVA detected significant differences between mean values, means were compared using Duncan’s test (*p* < 0.05).

## 3. Results

### 3.1. Antioxidant Activity

To select proper organic solvents and germination times of broccoli sprouts for further analysis, antioxidant properties were tested on samples using different extract solvents, including 70% methanol, 70% ethanol, and hot water, and various sprouting times, comprising 3, 5, 8, 10, and 12 days old. Firstly, samples were tested for determinations of total phenolic content (TPC), total flavonoid content (TFC) and vitamin C content. As shown in Table 1, TPC, TFC, and vitamin C content were significantly different at different germination times, in which five and eight days old sprouts contained the highest levels of these compounds. In another aspect, sprouts extracted by 70% methanol and 70% ethanol, with no statistically significant variations, obtained notably higher TPC, TFC and vitamin C content than samples extracted by hot water.

Subsequently, total antioxidant activity of broccoli sprouts reflected their ability to inhibit the production of free radicals. DPPH and ABTS radical cation assays were used to evaluate free radical scavenging properties, while reducing power assay determined the ability to reduce ferric (III) iron to ferrous (II) iron [23]. The results are presented in Table 2. When comparing the different solvents, similar to total antioxidant compound analysis, 70% ethanol and 70% methanol exhibited considerably higher and stable antioxidant values than hot water. Regarding germination times, and as indicated by both DPPH radical scavenging and reducing power assays, the highest antioxidant activities were evident in three-day sprouts, followed by five-day and eight-day sprouts. Moreover, according to the ABTS radical cation assay, three-day and five-day sprouts, with no statistically significant variations, displayed highly active antioxidant capacities, whereas the lower values were generally found during germination.

All results, as presented in Table 1 and Table 2, showed high antioxidant activities of broccoli sprouts at different germination times. After a statistically detailed comparison, the five-day-old broccoli sprouts extracted by 70% ethanol (BSE) exhibited the highest and most stable values of antioxidant activity. Thus, this group was selected for further tests.

**Figure 1:**
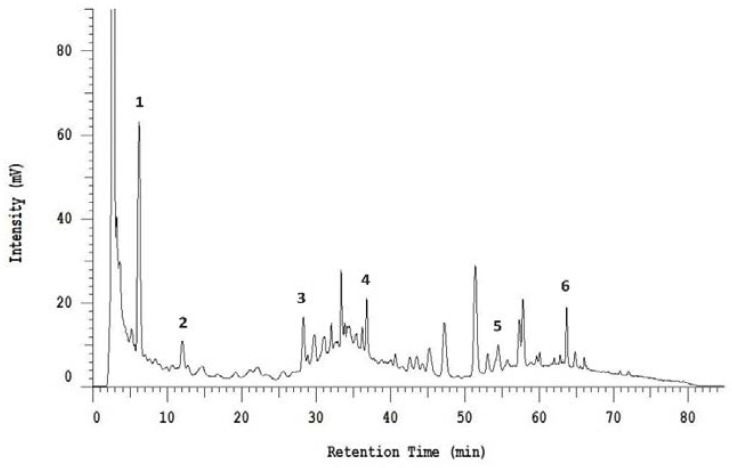
High-performance liquid chromatography (shown at 320 nm) indicating the phenolic profile of five-day-old sprout extract. Peaks: (1) Gallic acid, (2) esculetin, (3) caffeic acid, (4) ferulic acid, (5) myricetin, (6) quercetin.

Additionally, HPLC analysis revealed the presence of several phenolic acids and flavonoid derivatives in BSE. As shown in Figure 1, six compounds were identified, including gallic acid, esculetin, caffeic acid, ferulic acid, myricetin, and quercetin, which were analyzed to be 18.09, 214.99, 13.35, 264.27, 434.06, and 52.79 mg/100 g, respectively.

### 3.2. Antiproliferative and Apoptotic Activities

To study antiproliferative potential, five-day-old broccoli sprout extract (BSE) was evaluated against several cancer cell lines, including A549 (lung carcinoma cells), HepG2 (hepatocellular carcinoma cells) and Caco-2 (colorectal adenocarcinoma cells), but also tested on normal liver cells (FL83B), for potential selectivity. All cell lines were subjected to increasing concentrations of BSE (0.063–0.500 mg/mL) for 24 and 48 h. BSE induced cell death in a dose-dependent manner, and IC_50_ values (concentrations of extract leading to 50% inhibition of cell growth) were calculated. Table 3 shows that BSE exhibited considerable antiproliferative activity against the examined cancer cells, as indicated by the significant decrease in cell viability percentage. After 48 h of incubation, BSE was able to inhibit A549, HepG2, and Caco-2 cells with effective IC_50_ values of 0.117, 0.168, and 0.189 mg/mL, accordingly. In contrast, BSE actually did not affect the growth of normal liver cells, only a slight decrease of FL83B cell number was found. Cisplatin (positive control), used as a standard anticancer reference agent, presented the most effective IC_50_ values in comparison to BSE at all tested concentrations.

**Figure 2:**
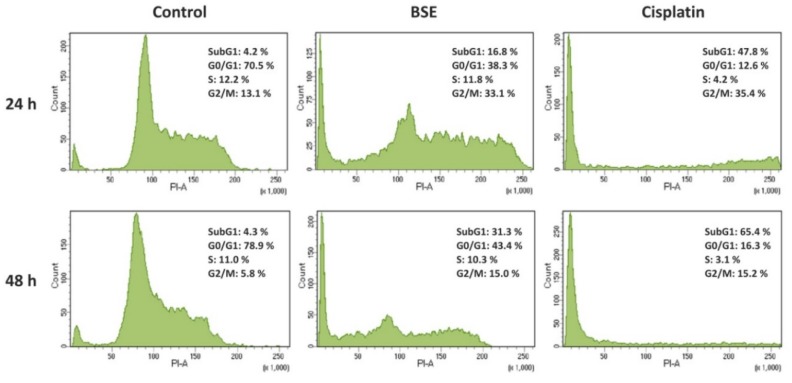
Effects of broccoli sprouts on Caco-2 cell cycle distribution. The percentage of Caco-2 cells at each phase of the cell cycle was analyzed by staining with PI using flow cytometry after treatment with or without sprout extract (BSE, 0.200 mg/mL) and cisplatin (0.020 mg/mL) for 24 and 48 h.

To further investigate the impact of BSE on the cancer cells, its apoptotic potential, which was considered to be associated with antiproliferative effect [1,13], was evaluated in Caco-2 cells by means of flow cytometry. Apoptosis was firstly characterized by cell cycle arrest and an increase of cell percentage at the subG1 phase in a time-dependent manner. As shown in Figure 2, the percentage of subG1 population in Caco-2 cells was significantly increased to 16.8 and 31.3% when exposed to BSE for 24 and 48 h, respectively, compared to approximately 4.2% of untreated cells. Cells treated with cisplatin demonstrated higher percentages at the subG1 phase after 24 and 48 h (47.8 and 65.4%, accordingly). Additionally, the substantial changes of cell populations at G0/G1 and G2 phases indicated cell cycle arrest after 48 h treatment by both BSE and cisplatin.

**Figure 3:**
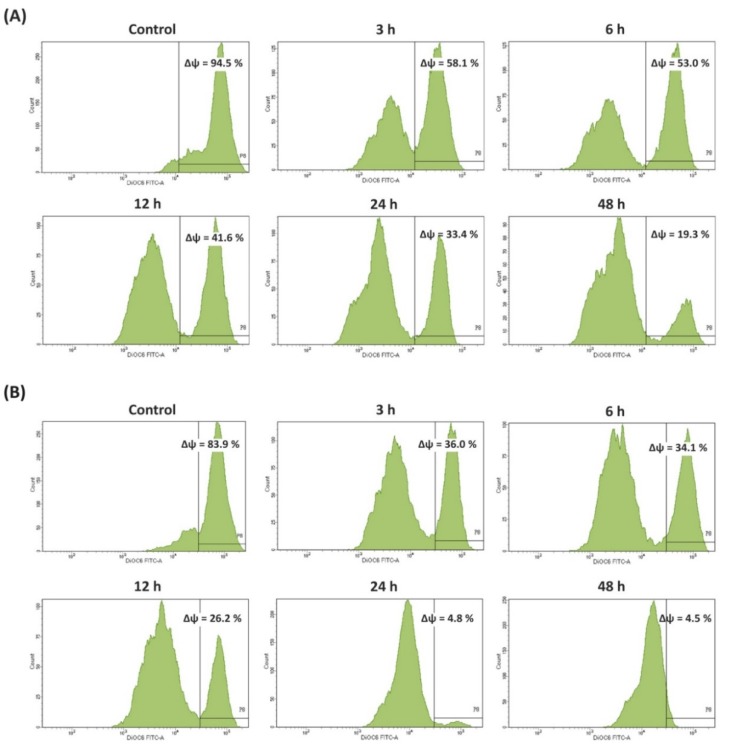
Effects of broccoli sprouts on mitochondrial membrane potential (MMP) in Caco-2 cells. The changes in the level of MMP were analyzed by staining with DiOC_6_ using flow cytometry after treatment with or without (**A**) sprout extract and (**B**) cisplatin for 3, 6, 12, 24, and 48 h.

Thereafter, apoptosis was also confirmed by loss of mitochondrial membrane potential (MMP) complemented with DiOC_6_ staining. As shown in Figure 3, an obvious decrease in MMP level was observed in BSE-treated Caco-2 cells in a time-dependent manner. The percentage of cell populations with disrupted MMP was 58.1, 53.0, 41.6, 33.4, and 19.3% after 3, 6, 12, 24, and 48 h of treatment with BSE, respectively, compared to the negative control (94.5%). The remarkable loss of MMP (to 4.5%) was also observed for cells exposed to cisplatin.

### 3.3. Antibacterial Activity

The antibacterial activities of BSE were qualitatively and quantitatively assessed by the presence or absence of inhibition zone diameters (IZD) and the minimum inhibitory concentration (MIC) values by the agar well diffusion method and the microdilution method, accordingly, against both Gram-positive (*Staphylococcus aureus* and *Bacillus subtilis*) and Gram-negative (*Escherichia coli* and *Salmonella* Typhimurium) bacterial strains. These bacteria are commonly known to cause food poisoning or food spoilage. In the case of plant crude extracts, the IZDs for antimicrobial activity were classified according to the following scale: Inhibition zones down to 12 mm as inactive, 12–15 mm as moderately active, 16–21 mm as active, and above 18 mm as highly active [26,30]. Additionally, plant extracts with MICs of approximately 0.5 mg/mL were indicative of high antimicrobial activity. MICs between 0.5 and 1.0 mg/mL were considered moderately active. MICs ranging from 1.0 to 8.0 mg/mL were classified to have weak activity, and MICs over 8.0 mg/mL were defined as inactive [30,32]. The results are presented in Table 4 and Table 5. BSE, at specific tested concentrations, showed significant antibacterial activities against the tested Gram-positive and Gram-negative bacteria with MIC values in the range of 0.78–1.56 mg/mL (IZD: 17.84–21.03 mm) and 0.39–1.56 mg/mL (IZD: 18.36–26.44 mm), respectively. The best activities were observed against *B. subtilis* with an MIC value of 0.39 mg/mL (IZD: 26.44 mm), followed by *S.* Typhimurium, *S. aureus*, and *E. coli*.

## 4. Discussion

Phenolic compounds and flavonoids are important groups of natural antioxidants for inclusion in the human diet, and some of them are also potential antimicrobial compounds. Therefore, it was essential to determine the total content of phenols and flavonoids in broccoli sprouts, which are known to be even richer in bioactive compounds than commercial broccoli florets [4,12]. Additionally, ascorbic acid has been directly associated with the modulation of plant growth, especially the early stage of embryo germination. Thus, vitamin C content in sprouts is favored by the development of the sprout, involving the reactivation of vitamin C biosynthesis [2,6]. Over the past years, numerous studies have analyzed the content of phenols [5,10,14], flavonoids [5,10,14] and vitamin C [2,6,22] of broccoli sprouts. Moreover, many extraction, separation and determination techniques such as HPLC, GC-MS, or LC-MS have been employed to obtain and characterize these antioxidant compounds from broccoli sprouts [5,9,10,12]. The high phenolic content along with the seed germination may influence free radical scavenging capacity, hence the antioxidant activity of broccoli sprouts have been studied in a number of publications using DPPH radical scavenging assay [4,5,7,18], ABTS radical cation assay [4,14,33], ferric reducing antioxidant power (FRAP) assay [4,5,14,18], or oxygen radical absorbance capacity (ORAC) assay [6,12]. This study also used varied analyses to confirm the antioxidant properties of broccoli sprouts extracted by different organic solvents at various germination times (Table 1 and Table 2 and Figure 1). Based on results, five-day-old broccoli sprouts extracted by 70% ethanol (BSE) were selected for further experiments, and such experiments strongly suggest its inclusion in the functional food industry. Interestingly, although the antioxidant potential of broccoli sprouts has been extensively studied, it is presently still obtaining considerable attention from food scientists and research groups [6,7,13].

In the past decade, cancer prevention by natural products has received considerable attention. The potentially protective role and active phytochemicals, such as phenols and glucosinolates (GLSs), of cruciferous vegetables have been extensively studied in in vitro and in vivo carcinogenesis models [12,34]. Broccoli sprouts have been recognized as a rich source of GLSs and related compounds (isothiocyanates, especially sulforaphane), and these hydrolytic products could reduce the risk of cancer by inducing apoptosis and arresting cell cycle progression [3,10]. Thus, to demonstrate the anti-cancerogenic effects of broccoli sprouts, a number of in vitro experiments have been undertaken to identify their antiproliferative activities [4,13,14,33,34] and to demonstrate their rich GLSs content [9,11,18,34]. The results of previous studies presented that broccoli sprouts achieved significant cytotoxic activity against various types of cancer cell lines, including MCF7 (breast adenocarcinoma), 786-O (renal adenocarcinoma), HT29 (colorectal adenocarcinoma), NCI-H460 (lung carcinoma), HepG2 (hepatocellular carcinoma cells), SW480 (colorectal adenocarcinoma), PC-3 (prostate carcinoma), Caco-2 (colorectal adenocarcinoma), and AGS (gastric adenocarcinoma) [4,13,14,33,34]. In these studies, the highly effective IC_50_ values were observed from 0.026 to 0.080 mg/mL. The results of this study also showed considerable antiproliferative activity against different cancer cell lines (Table 3), although they were not as effective as previous studies. Nonetheless, this study determined not only the cytotoxic potential but also the selectivity of BSE, whereby samples showed no effects on the viability of normal liver cells (FL83B). To the best of our knowledge, only a similar result was previously reported on normal skin fibroblasts (BJ) after the treatment of broccoli sprouts [13].

The molecular linkages between cell death, cell survival, and cell cycle have become an object of recent investigation. A lot of anticancer agents act by blocking stages of the cell cycle and eventually triggering apoptosis [1,3]. Therefore, to confirm whether the cytotoxicity was accompanied by apoptosis induction, our study revealed cell cycle arrest (obviously at G0/G1 phase) and the remarkable increase of cell number with subG1 DNA content, which is considered a marker of apoptosis (Figure 2). Moreover, mitochondrial changes induced by several stimuli activate the intrinsic apoptotic pathway. Loss of MMP due to membrane dysfunction leads to the activation of several proteins associated with programmed cell death, for example, pro/antiapoptotic proteins, cytochrome *c*, apoptosis inhibitors and activators [27,28]. Our result showed a notable decrease in the MMP after treatment with BSE (Figure 3). Only a few earlier reports documented broccoli sprouts’ effect on the apoptosis induction in cancer cells or the mechanism of cell death [7,13]. Thus, we can focus on the underlying molecular mechanism in both in vitro and in vivo models for future works. For instance, earlier research, tested in transgenic mouse models, proposed that broccoli sprouts could be effective for breast cancer prevention through modulation of epigenetic mechanisms [35].

Many plant extracts, rich in health-promoting compounds, such as phenolics, flavonoids, glucosinolates, and essential minerals, have shown to have high antimicrobial activity in vitro [19,36]. However, most of the previous research that focused on the biological activities of broccoli was related to antioxidant and anticancer properties. To date, although antimicrobial activities of broccoli florets have been the subject in several publications [15,16,17], no research has yet been reported on those of broccoli sprouts to foodborne pathogens. There is only a study that demonstrated the excellent bactericidal activity of broccoli sprouts against *Helicobacter pylori* strain [37]. Thus, the antimicrobial activities of broccoli florets and stems were discussed instead of sprouts. Previous studies overall confirmed the significant detrimental effects of broccoli floret extracts on pathogenic bacteria [15,16,17]. Comparatively, the MIC values (under 500 μg/mL) of floret extracts in those studies were more effective than that of BSE (Table 4) in this study. Moreover, some comparative studies have been conducted on the antibacterial activity of common vegetables, such as cauliflower, okra, carrot, and white cabbage against food spoilage and food pathogenic bacteria. Broccoli florets and cauliflower displayed higher antibacterial activities than the other vegetables tested [17,36]. Additionally, this study presented that *B. subtilis* was sensitive to BSE at a low concentration (0.39 mg/mL) and also showed a highly active IZD (26.44 mm), while *S.* Typhimurium and *E. coli* could be inhibited by BSE at higher MICs (0.78 and 1.56 mg/mL) and smaller IZDs (21.03 and 17.84 mm). These results are indicative that the Gram-positive bacteria were more sensitive to BSE than the Gram-negative bacteria, similar to earlier studies of other plant extracts. The possible reason could be due to the structural difference of Gram-positive and Gram-negative bacteria [30,31]. In addition to MIC and IZD, the mechanisms involved in the antimicrobial effects of BSE are worthy of further investigations. For example, a study showed that using flow cytometry to evaluate the antimicrobial activity of broccoli florets was very suitable and effective, enabling the identification of damaged and dead cells probably because of cell disruption and leakage of internal contents when submitted to the process of extract samples action [38].

## 5. Conclusions

Five-day-old broccoli sprout extract (BSE) showed the highest antioxidant activity at various germination times. BSE also proved to have considerable antiproliferative effects on different cancer cell lines, without toxic impact on normal cells, and the mechanism of cell death was associated with apoptosis induction after treatment with BSE. Moreover, notable antibacterial activities of BSE against tested foodborne bacteria were demonstrated for the first time. Therefore, these findings suggest that BSE could be considered as a valuable source of functional foods or pharmaceutical products due to its potential bioactivities and health-promoting benefits, or it could even be applied in pre-clinical studies as cancer chemopreventive agents.

Further investigations are required for exploring the detailed mechanisms of anticancer and antimicrobial properties of BSE in both in vitro and in vivo models, as well as other biological activities such as anti-inflammatory and enzyme inhibitory potential.

## Figures and Tables

**Table 1:** Antioxidant properties of broccoli sprouts at different germination times.

Germination Time	Total Phenolic Content (mg GAE/g DW)	Total Flavonoid Content (mg CE/g DW)	Vitamin C Content (mg AA/g DW)
Day 3			
70% Methanol	24.21 ± 1.07 ^cA^	5.10 ± 0.42 ^dA^	3.39 ± 0.28 ^bA^
70% Ethanol	20.72 ± 1.86 ^cB^	4.68 ± 0.15 ^dB^	2.88 ± 0.34 ^bAB^
Hot water	21.08 ± 1.59 ^aB^	4.35 ± 0.26 ^cB^	2.48 ± 0.31 ^aB^
Day 5			
70% Methanol	27.44 ± 0.37 ^aA^	6.08 ± 0.26 ^cA^	3.62 ± 0.14 ^abA^
70% Ethanol	29.27 ± 2.12 ^aA^	5.57 ± 0.22 ^cB^	3.83 ± 0.12 ^aA^
Hot water	23.31 ± 2.17 ^aB^	4.88 ± 0.13 ^bC^	2.63 ± 0.28 ^aB^
Day 8			
70% Methanol	27.07 ± 0.78 ^aB^	7.26 ± 0.26 ^aA^	3.59 ± 0.14 ^abA^
70% Ethanol	28.60 ± 0.80 ^aA^	7.30 ± 0.34 ^aA^	3.78 ± 0.26 ^aA^
Hot water	21.75 ± 1.27 ^aC^	4.96 ± 0.12 ^bB^	2.62 ± 0.23 ^aB^
Day 10			
70% Methanol	25.83 ± 0.63 ^bA^	6.68 ± 0.32 ^bA^	3.79 ± 0.30 ^abA^
70% Ethanol	23.54 ± 1.13 ^bB^	6.83 ± 0.26 ^bA^	3.29 ± 0.28 ^abAB^
Hot water	17.70 ± 1.48 ^bC^	5.67 ± 0.11 ^aB^	2.91 ± 0.25 ^aB^
Day 12			
70% Methanol	22.59 ± 0.89 ^dA^	5.90 ± 0.13 ^cB^	4.04 ± 0.35 ^aA^
70% Ethanol	21.53 ± 1.15 ^cA^	7.05 ± 0.09 ^abA^	3.31 ± 0.28 ^abA^
Hot water	12.90 ± 0.49 ^cB^	4.85 ± 0.10 ^bC^	2.43 ± 0.32 ^aB^

GAE, gallic acid equivalent; CE, catechin acid equivalent; AA, ascorbic acid equivalent; DW, dried weight. Values are expressed as mean ± SD of triplicate experiments. Mean values followed by different letters differ significantly (*p* < 0.05), the lowercase letters were presented for germination times, and the uppercase letters were presented for solvents.

**Table 2:** Antioxidant activity of broccoli sprouts at different germination times.

Germination Time	DPPH Scavenging Activity (Inhibition %)	Reducing Power Absorbance (700 nm)	ABTS Scavenging Activity (μmol TE/g DW)
Day 3			
70% Methanol	88.06 ± 1.61 ^aA^	1.90 ± 0.03 ^aB^	68.60 ± 0.13 ^aB^
70% Ethanol	91.90 ± 0.67 ^aA^	1.96 ± 0.01 ^aA^	69.10 ± 0.13 ^aA^
Hot water	76.24 ± 5.35 ^aB^	1.48 ± 0.07 ^aC^	65.91 ± 0.64 ^aC^
Day 5			
70% Methanol	83.02 ± 0.58 ^bB^	1.73 ± 0.04 ^bB^	68.56 ± 0.17 ^aA^
70% Ethanol	90.59 ± 0.44 ^aA^	1.81 ± 0.02 ^bA^	68.81 ± 0.25 ^abA^
Hot water	56.57 ± 4.57 ^bC^	1.00 ± 0.05 ^bC^	65.83 ± 0.55 ^aB^
Day 8			
70% Methanol	82.38 ± 0.53 ^bB^	1.71 ± 0.02 ^bA^	68.04 ± 0.27 ^bA^
70% Ethanol	88.82 ± 1.27 ^aA^	1.70 ± 0.01 ^cA^	68.61 ± 0.23 ^bA^
Hot water	55.09 ± 0.86 ^bC^	1.00 ± 0.06 ^bB^	65.06 ± 0.99 ^aB^
Day 10			
70% Methanol	75.43 ± 2.62 ^cB^	1.60 ± 0.02 ^cA^	66.65 ± 0.27 ^cB^
70% Ethanol	83.80 ± 2.79 ^bA^	1.58 ± 0.02 ^dB^	68.53 ± 0.20 ^bA^
Hot water	41.11 ± 2.71 ^cC^	0.49 ± 0.03 ^cC^	61.94 ± 1.31 ^bC^
Day 12			
70% Methanol	69.46 ± 3.13 ^dB^	1.47 ± 0.02 ^dB^	65.73 ± 0.42 ^dB^
70% Ethanol	81.27 ± 2.89 ^bA^	1.50 ± 0.05 ^eA^	67.46 ± 0.68 cA
Hot water	40.04 ± 4.87 ^cC^	0.40 ± 0.02 ^dC^	59.60 ± 0.97 ^cC^
Ascorbic acid *	96.31 ± 1.08	2.59 ± 0.04	66.86 ± 0.21

DPPH, 2,2-diphenyl-1-picrylhydrazyl; ABTS, 2,2′-azino-bis-3-ethylbenzothiazoline-6-sulphonic; TE, Trolox equivalent; DW, dried weight. Values are expressed as mean ± SD of triplicate experiments. Mean values followed by different letters differ significantly (*p* < 0.05), the lowercase letters were presented for germination times, and the uppercase letters were presented for solvents. * Positive control used (0.5 mg/mL).

**Table 3:** Antiproliferative activity of broccoli sprouts against different cell lines.

Cell Lines	Cell Growth (%) in Different BSE Concentrations (mg/mL)				IC50 (mg/mL) ^a^	
	0.063	0.125	0.250	0.500	BSE	Cisplatin ^b^
A549						
24 h	68.31 ± 4.14	61.35 ± 3.09	45.09 ± 4.76	39.57 ± 3.61	0.226 ± 0.022	0.009 ± 0.001
48 h	61.12 ± 6.98	47.56 ± 9.32	34.72 ± 3.04	26.40 ± 2.19	0.117 ± 0.026	<0.006
HepG2						
24 h	84.63 ± 8.86	80.76 ± 7.09	57.97 ± 5.62	40.63 ± 6.00	0.355 ± 0.033	0.019 ± 0.001
48 h	70.21 ± 4.41	55.76 ± 5.56	39.07 ± 6.60	31.58 ± 4.27	0.168 ± 0.019	0.015 ± 0.003
Caco-2						
24 h	75.98 ± 5.79	70.36 ± 6.08	56.02 ± 3.27	41.89 ± 2.61	0.338 ± 0.019	0.023 ± 0.002
48 h	69.51 ± 4.94	57.35 ± 5.83	45.15 ± 3.82	34.96 ± 3.25	0.189 ± 0.013	0.007 ± 0.003
FL83B						
24 h	113.89 ± 3.85	115.56 ± 3.95	103.69 ± 6.54	104.12 ± 7.29	>0.500	>0.050
48 h	103.45 ± 9.10	99.96 ± 7.44	94.69 ± 8.96	96.90 ± 6.20	>0.500	0.027 ± 0.005

^a^ IC_50_, the concentration of compound that affords a 50% reduction in cell growth (after 24 and 48 h incubation); BSE, five-day-old sprout extract; ^b^ Cisplatin, positive control (0.006–0.050 mg/mL); Expressed as the mean ± SD of triplicate experiments.

**Table 4:** Antibacterial activity of broccoli sprouts.

Microorganism	Diameter of the Inhibition Zones (mm) ^a^			
	BSE	Amp	Amo	D20
Gram-positive				
*Staphylococcus aureus*	18.36 ± 1.61	36.38 ± 0.76	33.76 ± 0.56	ND
*Bacillus subtilis*	26.44 ± 1.07	38.10 ± 0.75	37.33 ± 1.39	ND
Gram-negative				
*Escherichia coli*	17.84 ± 0.77	39.04 ± 0.87	36.00 ± 0.60	ND
*Salmonella* Typhimurium	21.03 ± 0.34	43.54 ± 0.19	40.84 ± 0.47	ND

^a^ The diameter of the inhibition zones (mm), including the well diameter (9 mm), are given as the mean ± SD of triplicate experiments. Diameter of the inhibition zones of broccoli sprout extract (BSE, 50 mg/mL), positive control: Amp, ampicillin and Amo, amoxicillin (0.1 mg/mL), and negative control: D20, 20% DMSO. ND, not detected.

**Table 5:** Minimum inhibitory concentration (MIC) of broccoli sprouts.

Microismorgan	Concentration (mg/mL)										NT	Amp
	0.10	0.2	0.39	0.78	1.56	3.13	6.25	12.5	25	50		
Gram-positive												
*Staphylococcus aureus*	+	+	+	+	−	−	−	−	−	−	+	−
*Bacillus subtilis*	+	+	−	−	−	−	−	−	−	−	+	−
*Gram-negative*												
*Escherichia coli*	+	+	+	+	−	−	−	−	−	−	+	−
*Salmonella* Typhimurium	+	+	+	−	−	−	−	−	−	−	+	−

+ Growth of microorganism; − No growth of microorganisms; Amp, Ampicillin (0.1 mg/mL); NT, not treated by antibiotic or extract, negative control.
